# Curcumin inhibits the growth of triple‐negative breast cancer cells by silencing EZH2 and restoring DLC1 expression

**DOI:** 10.1111/jcmm.15683

**Published:** 2020-07-28

**Authors:** Xueliang Zhou, Dechao Jiao, Mengmeng Dou, Weijie Zhang, Liying Lv, Jianjian Chen, Lifeng Li, Liuxing Wang, Xinwei Han

**Affiliations:** ^1^ Department of Interventional Radiology The First Affiliated Hospital of Zhengzhou University Zhengzhou China; ^2^ Department of Neurology The First Affiliated Hospital of Zhengzhou University Zhengzhou China; ^3^ Department of Oncology The First Affiliated Hospital of Zhengzhou University Zhengzhou China; ^4^ Department of Oncology The Central Hospital of Kaifeng Kaifeng China

**Keywords:** breast cancer, curcumin, DLC1, EZH2

## Abstract

Enhancer of zeste homolog 2 (EZH2), an oncogene, is a commonly up‐regulated epigenetic factor in human cancer. Hepatocellular carcinoma deletion gene 1 (DLC1) is an antioncogene that is either expressed at low levels or not expressed in many malignant tumours. Curcumin is a promising anticancer drug that has antitumour effects in many tumours, but its mechanism of action is unclear. Our research demonstrated that EZH2 was up‐regulated in breast cancer (BC) tissues and cells, whereas DLC1 was down‐regulated, and the expression of EZH2 and DLC1 was negatively correlated in BC. By analysing the characteristics of clinical cases, we found that positive expression of EZH2 and negative expression of DLC1 may be predictors of poor prognosis in patients with triple‐negative breast cancer (TNBC). Moreover, knockdown of EZH2 expression restored the expression of DLC1 and inhibited the migration, invasion and proliferation, promoted the apoptosis, and blocked the cell cycle of MDA‐MB‐231 cells. Furthermore, we found that curcumin restored the expression of DLC1 by inhibiting EZH2; it also inhibited the migration, invasion and proliferation of MDA‐MB‐231 cells, promoted their apoptosis and blocked the cell cycle. Finally, xenograft tumour models were used to demonstrate that curcumin restored DLC1 expression by inhibiting EZH2 and also inhibited the growth and promoted the apoptosis of TNBC cells. In conclusion, our results suggest that curcumin can inhibit the migration, invasion and proliferation, promote the apoptosis, block the cycle of TNBC cells and restore the expression of DLC1 by inhibiting the expression of EZH2.

## 1 INTRODUCTION

Breast cancer (BC), one of the most common cancers in women, accounts for approximately 30% of new tumours in women and is also the main cause of cancer‐related death.^1^ In 2018, the number of newly diagnosed female BC cases was estimated to be 2.1 million worldwide, accounting for nearly a quarter of female cancer cases, and its incidence has been on the rise in countries such as Asia, Africa and South America.^2^ The increased risk of BC is closely related to several important factors, including increased age, number of first‐degree relatives with BC, atypical hyperplasia and age of menarche.^3^ As a molecular subtype of BC, triple‐negative breast cancer (TNBC) lacks the expression of ER, PR and HER2.^4^ The prognosis of patients with TNBC is worse than that of non‐TNBC patients. Non‐TNBC patients may benefit from the anti‐HER2 antibody trastuzumab and endocrine therapy.^5^, ^6^ Studies have found that the release of upstream epigenetic regulatory factors can promote epigenetic changes, leading to abnormal silencing of antioncogenes, which is also an important mechanism for promoting cancer.^7^

Drosophila zeste gene enhancer homologue 2 (EZH2), as a catalytic subunit of PRC2, is a commonly up‐regulated epigenetic factor in cancer.^8^ As a histone methyltransferase, EZH2 can specifically catalyse histone H3K27me3 and inhibit histone modification to control epigenetic transcriptional regulation.^9^ Therefore, the up‐regulation of EZH2 can promote the metastasis of cancers and play a pivotal role in the progression of cancers.^10^

Hepatocellular carcinoma deletion gene 1 (DLC1), an antioncogene, is located on human chromosome 8p22 and is either expressed at low levels or not expressed in 50% of human hepatocellular carcinomas and many other human cancers (including colon cancer, lung cancer, prostate cancer and BC).^11^, ^12^, ^13^ In addition to DLC1, 8p22 also contains other tumour suppressor genes, such as MTUS1, TUSC35 and FGL1.^14^ Moreover, in addition to genomic deletion in tumours, down‐regulation of DLC1 expression and promoter methylation is also common in human tumours, which makes DLC1 the most important tumour suppressor on 8p22.^11^, ^15^

A large number of studies have proven that curcumin has strong anti‐inflammatory, antioxidant and antitumour properties.^16^, ^17^ In pancreatic cancer, curcumin has been known to inhibit many oncogenes, including VEGF, Akt, Erk, cytochrome c oxidase subunit II and EZH2.^18^ EZH2 has been considered as a target for curcumin in pancreatic and colorectal cancer.^19^, ^20^

EZH2 is an oncogene, and DLC1 is an antioncogene, and both of them are related to epigenetics. The objective of our study was to investigate the regulatory relationship between EZH2 and DLC1 in BC and to determine whether curcumin can inhibit the growth of TNBC cells by silencing EZH2 to restore the expression of DLC1.

## 2 MATERIALS AND METHODS

### 2.1 Analysis of gene differential expression and correlation based on TCGA database

UALCAN (http://ualcan.path.uab.edu/) and GEPIA (http://gepia.cancer‐pku.cn/), two‐level online analysis tools based on TCGA, were used to analyse the differential expression and correlation of EZH2 and DLC1 in BC and a variety of other tumours, and further stratified analyses were conducted on the expression of EZH2 and DLC1 in patients with different clinical characteristics.

### 2.2 Clinical samples

From January 2012 to December 2013, 100 pairs of BC tissue and adjacent normal breast tissue were collected from 100 patients who underwent surgical resection and pathological confirmation in the First Affiliated Hospital of Zhengzhou University. None of the patients received chemotherapy or radiotherapy before the operation. The Medical Research Ethics Committee of the First Affiliated Hospital of Zhengzhou University approved this study. Samples were used for quantitative real‐time polymerase chain reaction (qRT‐PCR), Western blotting (WB) analysis and immunohistochemistry (IHC) staining. The clinical characteristics of BC patients were collected and analysed, including age, tumour size, status of lymph node metastasis, TNM tumour stage and molecular subtype. The classification of TNM of BC was established by the Joint American Cancer Commission (AJCC).^21^

### 2.3 Cell culture, transfection and drug treatment

The normal breast cell line (HBL 100) and BC cell lines (MCF‐7, MDA‐MB‐231 and MDA‐MB‐468) were purchased from the Chinese Academy of Sciences Type Culture Collection. The HBL 100, MCF‐7 and MDA‐MB‐231 cell lines were inoculated in DMEM (containing 10% foetal bovine serum, 1% penicillin and 1% streptomycin). The MDA‐MB‐468 cells were inoculated in 1640 medium (containing 10% foetal bovine serum, 1% penicillin and 1% streptomycin). All cells were cultured in an incubator at 37°C with a 5% CO_2_ volume fraction.

The siRNA oligonucleotide of EZH2 and the negative control were synthesized by Gemma Gene Company. MDA‐MB‐231 cells were transfected with Lipofectamine 3000 reagent (Invitrogen) and Opti MEM (Gibco) according to the manufacturer's instructions. MDA‐MB‐231 cells were inoculated into 6‐well plates. When the cell growth density reached 70%‐80%, the culture medium in the 6‐well plates was discarded, and the complexes prepared with Lipofectamine 3000, Opti‐MEM and siRNA were added.

EPZ‐6438 (MedChemExpress) was used as an EZH2 inhibitor to treat cells at a concentration of 2 μmol/L for 7 days, and then cells were collected for subsequent experiments. Curcumin (Sigma) was dissolved in DMSO, and the cells were treated with concentrations of 20 μmol/L and 40 μmol/L.

### 2.4 Immunohistochemistry

The main antibodies were EZH2 (1:50, CST) and DLC1 (1:50, Abcam). The intensity of the immune response was evaluated according to the percentage of positive cells in sections. The grading criteria were as follows: 0, no staining; 1, 1%‐25% positive cells; 2, 25%‐50% positive cells; 3, 50%‐75% positive cells; and 4, >75% positive cells. Tumours with grades greater than or equal to grade 2 are considered positive for antigen expression.

### 2.5 RNA extraction and qRT‐PCR

Total RNA was extracted by TRIzol reagent (TaKaRa), and cDNA was synthesized by reverse transcription using PrimeScript™ RT Master Mix (TaKaRa) according to the manufacturer's instructions. The levels of GAPDH, DLC1 and EZH2 were detected by qRT‐PCR using SYBR@ Premix Ex TaqTM (Roche). The results were normalized to the expression of GAPDH. The primer sequences were synthesized by Sangon (Table 1). The relative expression levels of EZH2 and DLC1 were quantitatively calculated by the 2^(−ΔΔCT)^ method.

**TABLE 1:** The list of primers and siRNA sequence

GENE	Forward primer	Reverse primer
EZH2	AGGAGTTTGCTGCTGCTCTC	CCGAGAATTTGCTTCAGAGG
DLC1	CACAGGACAACCGTTGCCTCGA	CTCTTCAGGGTGTTGAGATGGA
GAPDH	TTGGTATCGTGGAAGGACTCA	TGTCATCATATTTGGCAGGTT
Sequences for siRNAs		
si‐EZH2‐1#	GACUCUGAAUGCAGUUGCUTT	AGCAACUGCAUUCAGAGUCTT
si‐EZH2‐2#	GCUCCUCUAACCAUGUUUATT	UAAACAUGGUUAGAGGAGCTT

### 2.6 Western blot assay and antibodies

Total proteins were extracted with RIPA lysate containing protease inhibitors. Protein was isolated by SDS‐PAGE. The protein bands were transferred to PVDF membranes after electrophoresis. After blocking with 5% skimmed milk powder, the primary antibody was added, and the reaction lasted overnight at 4°C. Immunoblots were performed with the following primary antibodies: rabbit anti‐EZH2 (1:1000, CST), rabbit anti‐DLC1 (1:1000, Abcam), rabbit anti‐H3K27me3 (1:1000, Abcam), rabbit anti‐CDK1 (1:30 000, Servicebio), rabbit anti‐caspase‐9 (1:1000, Servicebio), rabbit anti‐Bcl‐2 (1:1000, Sanying), rabbit anti‐cyclin A1 (1:1000, Abclonal), rabbit anti‐β‐actin (1:1000, Sanying) and mouse anti‐GAPDH (1:1000, Santa Cruz). GAPDH or β‐actin antibody was employed as a control. Afterwards, the membrane was incubated with the secondary antibody for 1 hour. ECL detection reagent (Santa Cruz) was used to detect the signal. ImageJ software was used to analyse the grey values of the protein bands.

### 2.7 Chromatin immunoprecipitation (ChIP) assay

ChIP assays were performed using a ChIP kit (Cell Signaling Technology, USA) according to the manufacturer's protocol. ChIP‐grade antibody against H3K27me3 (Abcam, USA) was used in the assay. Rabbit IgG (Cell Signaling Technology, USA) was used as a negative control in the assay. Precipitated DNA samples were analysed by PCR with primer pairs specific for the promoters of the DLC1 gene. The following primers were used: DLC1, sense primer, 5'‐GGGATTTTTGAGATCACTCTTAGG‐3', anti‐sense primer, 5'‐CTGGAATACAACAACTTTGCACC‐3'. The fold enrichment from the ChIP assay was calculated with reference to the IgG control after normalization to the input DNA. Each experiment was repeated three times.

### 2.8 Proliferation assay

MDA‐MB‐231 cells (3000 cells/well) from the experimental and control groups were seeded in 96‐well plates. The cell viability at 24, 48 and 72 hour was measured by CCK‐8 assay. The cell proliferation ability was expressed as the absorbance ratio of the experimental group and the control group at 450 nm.

### 2.9 Cell apoptosis assay

The Annexin V‐FITC/PI Apoptosis Detection Kit (BD) was used to detect the apoptosis of cells by flow cytometry according to the manufacturer's instructions. The cells were collected and resuspended in buffer, stained in the dark and analysed by flow cytometry.

### 2.10 Cell cycle assay

A cell cycle assay kit (KeyGEN, China) was used to assess cell cycle distribution by flow cytometry according to the manufacturer's instructions. The cells were collected and fixed overnight with 500 µL of 70% cold ethanol. Finally, the cell cycle assay kit was used for staining, and the results were analysed by flow cytometry.

### 2.11 Cell migration and invasion assays

A wound healing assay was used to detect cell migration. First, the cells were inoculated into 6‐well plates and cultured to 80% confluence. Then, a 200‐microlitre sterile pipet tip was used to draw a line across the central area of cell growth, and pictures were taken at 0 and 48 hours after scratching. The Transwell method was used to detect cell migration and invasion. The cells were suspended in serum‐free medium and inoculated in a 24‐well Transwell plate (Corning), and DMEM containing 20% foetal bovine serum was added as a chemotactic agent in the lower chamber. The uninvaded cells were wiped off with a cotton swab after 12 hours of culture. The cells were fixed with 4% paraformaldehyde for 30 minutes, stained with crystal violet and counted under a microscope. On the basis of the above procedure, Matrigel (BD Biosciences) was diluted and placed in the upper chamber of a 24‐well Transwell plate for the invasion assay.

### 2.12 Tumour xenograft experiments

MDA‐MB‐231 cells (5 × 10^6^/200 μL/site) at the logarithmic growth stage were inoculated into the right groin of 5‐week‐old female BALB/c nude mice. The formula: volume (mm^3^) = (length × width^2^)/2 was used to calculate tumour volume. Nude mice were randomly divided into two groups (5/group) after 7 days. The experimental group was treated with curcumin (100 mg/kg/2 days), and the control group was given an equal volume of PBS. Tumour volume was measured every three days, and the tumour was removed and weighed after 28 days. The animal experiments were approved by the Ethics Review Committee of Zhengzhou University.

### 2.13 Statistical analysis

Statistical analysis was performed using SPSS 17.0 (SPSS, Inc, USA) and GraphPad Prism 5 (GraphPad Software Inc, San Diego, CA, USA) in this study. The data among the groups were analysed by t test or one‐way analysis of variance (ANOVA) using the mean ± standard deviation. The chi‐square test was used to analyse the difference and correlation between clinicopathological parameters, and the Kaplan‐Meier chart and log rank test were used for survival analysis. The difference was statistically significant with *P* < 0.05.

## 3 RESULTS

### 3.1 The expression of EZH2 is up‐regulated and DLC1 is down‐regulated in various tumours

**FIGURE 1:**
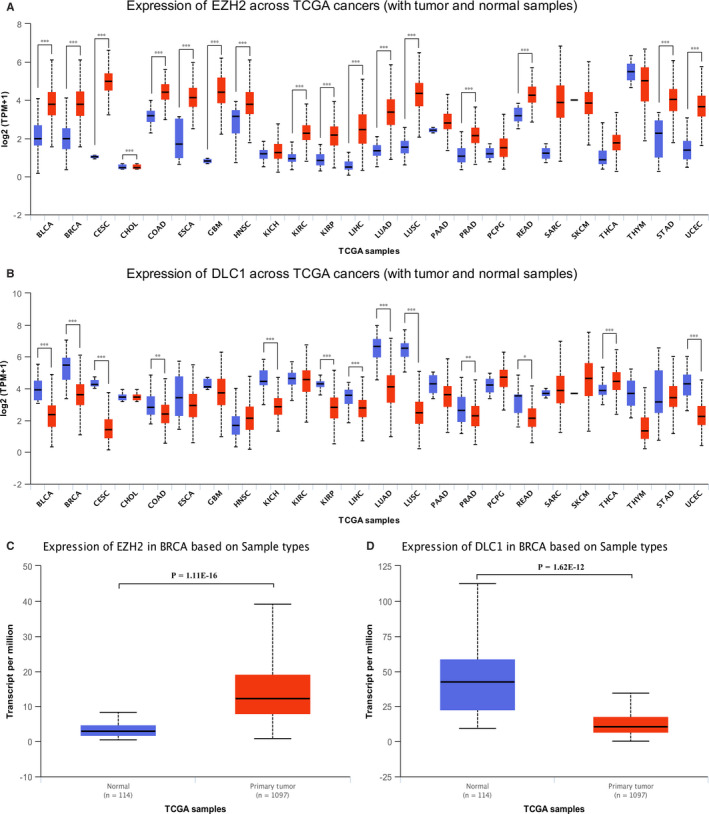
The expression of EZH2 and DLC1 in various tumours and normal tissues from the UALCAN database. A, EZH2 is highly expressed in a variety of tumours. B, DLC1 is expressed at low levels in a variety of tumours. C, Expression of EZH2 in breast cancer tissues and normal breast tissues. D, Expression of DLC1 in breast cancer tissue and normal breast tissue. BLCA, Bladder urothelial carcinoma; BRCA, Breast invasive carcinoma; CESC, Cervical squamous cell carcinoma; CHOL, Cholangiocarcinoma; COAD, Colon adenocarcinoma; ESCA, Oesophageal carcinoma; GBM, Glioblastoma multiforme; HNSC, Head and Neck squamous cell carcinoma; KICH, Kidney Chromophobe; KIRC, Kidney renal clear cell carcinoma; KIRP, Kidney renal papillary cell carcinoma; LIHC, Liver hepatocellular carcinoma; LUAD, Lung adenocarcinoma; LUSC, Lung squamous cell carcinoma; PAAD, Pancreatic adenocarcinoma; PRAD, Prostate adenocarcinoma; PCPG, Pheochromocytoma and Paraganglioma; READ, Rectum adenocarcinoma; SARC, Sarcoma; SKCM, Skin cutaneous melanoma; THCA, Thyroid carcinoma; THYM, Thymoma; STAD, Stomach adenocarcinoma; UCEC, Uterine corpus endometrial carcinoma; *P < 0.05; **P < 0.01; ***P < 0.001

To explore the expression of EZH2 and DLC1 in tumours, we analysed the expression of EZH2 and DLC1 in various tumours based on the secondary online tools of the TCGA database. The results showed that the expression of EZH2 was up‐regulated in various tumours (liver hepatocellular carcinoma, bladder urothelial carcinoma, colon adenocarcinoma, etc) compared with normal tissues (Figure 1A). In contrast, the expression of DLC1 was down‐regulated in various tumours (liver hepatocellular carcinoma, bladder urothelial carcinoma, colon adenocarcinoma, etc) compared with normal tissues (Figure 1B). EZH2 expression was also up‐regulated and DLC1 expression was down‐regulated in BC tissue compared with normal breast tissue (Figure 1C,D).

### 3.2 EZH2 expression is up‐regulated and DLC1 is down‐regulated in BC tissues and cell lines

**FIGURE 2:**
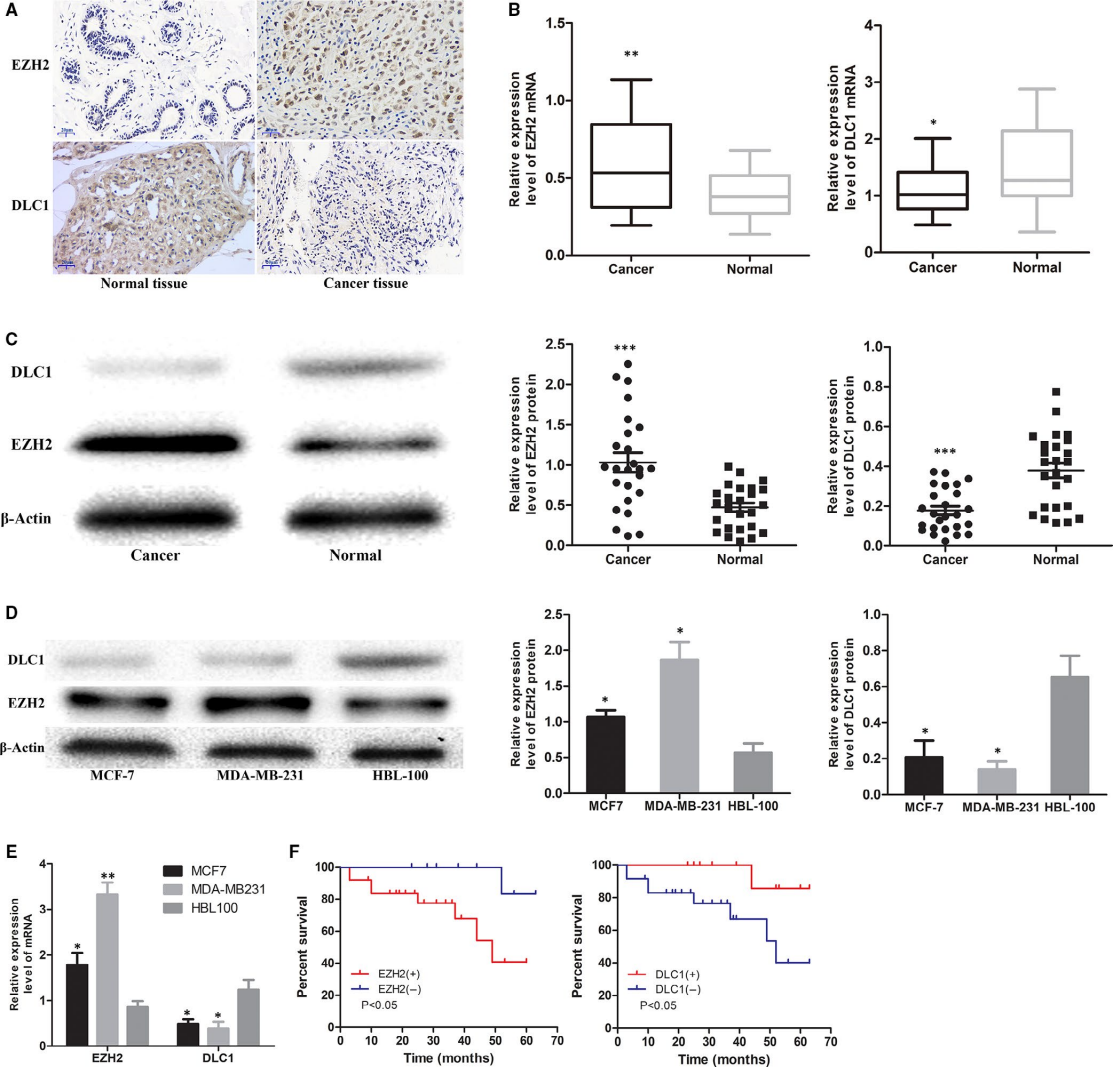
Expression of EZH2 and DLC1 in breast cancer tissues and cell lines. A, Representative pictures of EZH2 and DLC1 immunohistochemical (IHC) staining in breast cancer and adjacent normal breast tissues (×400, bar = 20 μm, n = 100). B, qRT‐PCR analysis showing the relative transcription of EZH2 and DLC1 in breast cancer and adjacent normal breast tissues (n = 25). C, Expression of EZH2 and DLC1 protein in breast cancer and adjacent normal breast tissues (n = 25). D, Expression of EZH2 and DLC1 protein in human breast cancer cell lines (MCF‐7 and MDA‐MB‐231) and a normal human breast cell line (HBL‐100). E, qRT‐PCR analysis showing the relative transcription of EZH2 and DLC1 in human breast cancer cell lines (MCF‐7 and MDA‐MB‐231) and a normal human breast cell line (HBL‐100). F, Kaplan‐Meier plot showing the overall survival of triple‐negative breast cancer (TNBC) patients with positive vs. negative EZH2 (left) or DLC1 (right) expression. *P < 0.05; **P < 0.01; ***P < 0.001

To further confirm the expression of EZH2 and DLC1 in BC, 100 pairs of BC tissues and matched normal tissues were examined. IHC results demonstrated that EZH2 was positive in 73% of BC tissues, while DLC1 was only positive in 36% of BC tissues. EZH2 was highly expressed in breast cancer tissues (t = 9.236, *P* = 0.000), while DLC1 was expressed at low levels in breast cancer tissues (t = −3.238, *P* = 0.002) (Figure 2A). WB and qRT‐PCR analysis showed that EZH2 was up‐regulated and DLC1 was down‐regulated in BC tissues (Figure 2B,C). Moreover, EZH2 expression was higher in BC cell lines (MCF‐7 and MDA‐MB‐231) than in normal breast cells (HBL‐100). Among these cell lines, EZH2 was the most highly expressed in MDA‐MB‐231 cells. However, the expression of DLC1 in MCF‐7 and MDA‐MB‐231 cells was lower than that in HBL‐100 cells and lowest in MDA‐MB‐231 cells (Figure 2D,E). These data indicate that EZH2 is up‐regulated in BC, whereas DLC1 is down‐regulated in BC.

### 3.3 EZH2 and DLC1 may be predictors of prognosis in patients with TNBC

**FIGURE 3:**
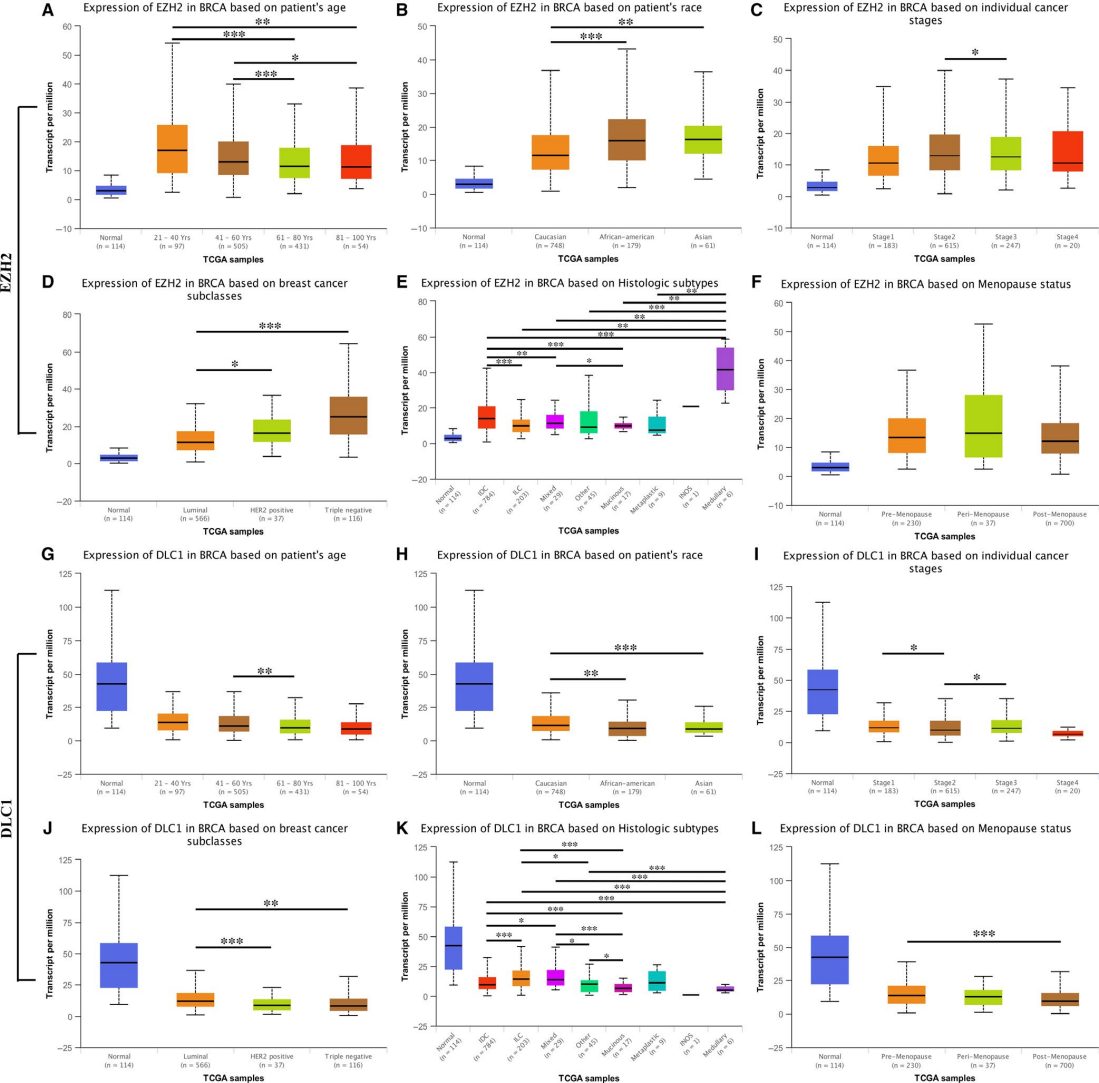
The relationship between EZH2, DLC1 and clinical characteristics of breast cancer patients from the UALCAN database. A, Expression of EZH2 in BRCA based on patient age. B, Expression of EZH2 in BRCA based on patient race. C, Expression of EZH2 in BRCA based on individual cancer stages. D, Expression of EZH2 in BRCA based on molecular subtypes. E, Expression of EZH2 in BRCA based on histologic subtypes. F, Expression of EZH2 in BRCA based on menopause status; G, Expression of DLC1 in BRCA based on patient age. H, Expression of DLC1 in BRCA based on patient race. I, Expression of DLC1 in BRCA based on individual cancer stages. J, Expression of DLC1 in BRCA based on molecular subtypes. K, Expression of DLC1 in BRCA based on histologic subtypes. L, Expression of DLC1 in BRCA based on menopausal status. BRCA: Breast invasive carcinoma. *P < 0.05; **P < 0.01; ***P < 0.001

To explore the relationship between EZH2 and DLC1 and the clinical characteristics of BC patients, we first carried out subgroup analysis based on the TCGA database. The results showed that EZH2 and DLC1 were differentially expressed in patients of different ages (Figure 3A,G), races (Figure 3B,H), cancer stages (Figure 3C,I), molecular subtypes (Figure 3D,J) and histologic subtypes (Figure 3E,K). The expression of DLC1 was also different between premenopausal and postmenopausal patients with BC (Figure 3L). In addition, as shown in Table 2, the positive expression of EZH2 is associated with lymph node metastasis (*P* = 0.02) and tumour size (*P* = 0.01), while the negative expression of DLC1 is associated with lymph node metastasis (*P* = 0.043) and tumour size (*P* = 0.036). Moreover, we found that TNBC patients with positive expression of EZH2 and negative expression of DLC1 had shorter overall survival (*P* < 0.05, Figure 2F).

**TABLE 2:** The association between the expression of EZH2 and DLC1 and clinicopathological features in breast cancer patients

Parameter	n	Expression of EZH2		χ^2^	*P*	Expression of DLC1		χ^2^	*P*
		Positive	Negative			Positive	Negative		
Age(year)									
<50	39	27	12	0.461	.497	13	26	0.197	.657
≥50	61	46	15			23	38		
Tumour size(cm)									
<2	29	16	13	6.586	.010	15	14	4.383	.036
≥2	71	57	14			21	50		
Lymph node metastasis									
Yes	37	32	5	5.420	.020	18	19	4.078	.043
No	63	41	22			18	45		
TNM tumour stage									
Ⅰ‐Ⅱ	79	60	19	1.660	.198	26	53	1.558	.212
Ⅲ‐Ⅳ	21	13	8			10	11		
Molecular subtype									
Luminal A	45	34	11	0.378	.539^a^	17	28	0.172	.678^b^
Luminal B	8	6	2			3	5		
HER2(+)	11	8	3			4	7		
Triple negative	36	25	11			12	24		

^a^ EZH2 expression in luminal A compared with Triple Negative.

^b^ DLC1 expression in luminal A compared with Triple Negative.

### 3.4 EZH2 regulates the expression of DLC1 in MDA‐MB‐231 cells by mediating H3K27me3

**FIGURE 4:**
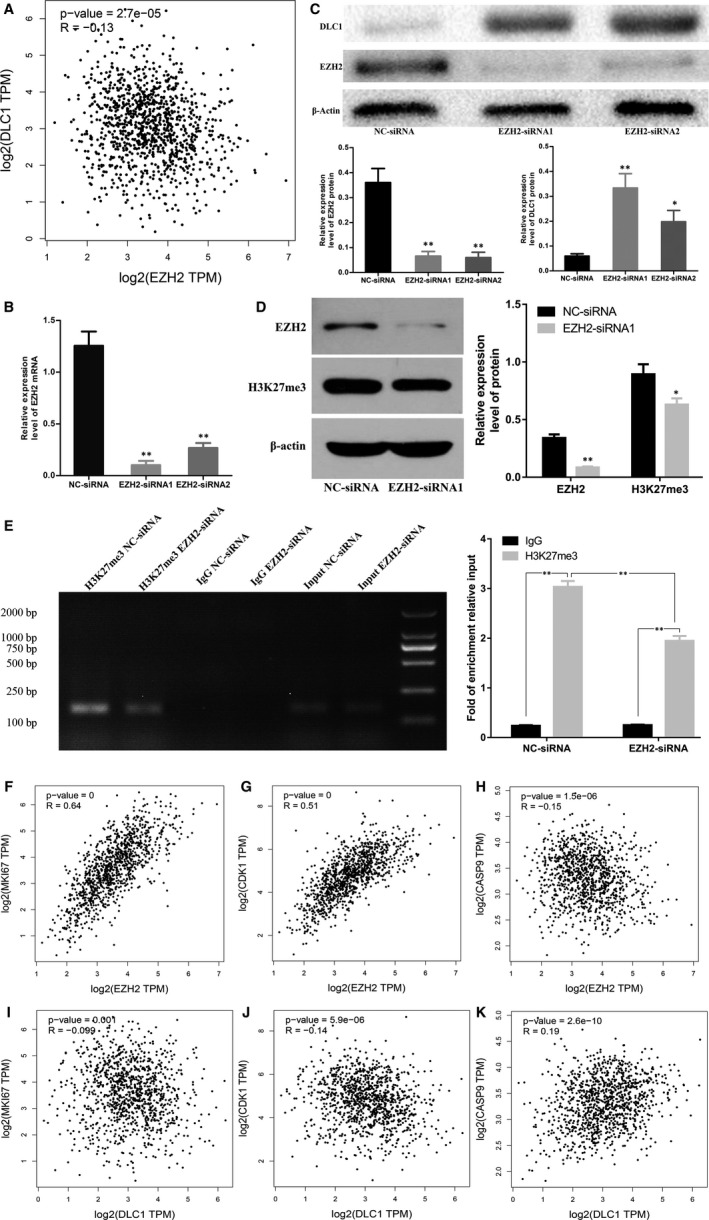
Knocking down EZH2 can restore the expression of DLC1 in MDA‐MB‐231 cells and correlation analysis of EZH2 or DLC1 genes based on TCGA database. A, EZH2 and DLC1 correlation analysis based on TCGA database. B, Expression of EZH2 in MDA‐MB‐231 cells after EZH2‐siRNA or NC‐siRNA transfection. C, Expression of EZH2 and DLC1 protein in MDA‐MB‐231 cells after EZH2‐siRNA or NC‐siRNA transfection. D, Knockdown of EZH2 can inhibit H3K27me3 expression. E, Chromatin immunoprecipitation (ChIP) assay coupled with PCR analysis revealed the relative enrichment of H3K27me3 in the DLC1 promoter region in MDA‐MB‐231 cells, while the knockdown of EZH2 reduced the enrichment of H3K27me3 in the promoter region of DLC1. The fold enrichment from the ChIP assay was calculated with reference to the IgG control after normalization to the input DNA. F, The correlation between EZH2 and MKI67. G, The correlation between EZH2 and CDK1. H, The correlation between EZH2 and CASP9. I, The correlation between DLC1 and MKI67. J, The correlation between DLC1 and CDK1. K, The correlation between DLC1 and CASP9. *P < 0.05; **P < 0.01

The results of correlation analysis based on the TCGA database showed that the expression levels of EZH2 and DLC1 were negatively correlated in BC (*R* = −0.13, *P* = 2.7 × 10^−5^, Figure 4A). Moreover, our correlation analysis also showed that the expression levels of EZH2 and DLC1 were negatively correlated in 100 BC tissues. (*R* = −0.248, *P* = 0.013). To further determine the relationship between EZH2 and DLC1, we used EZH2 siRNA or negative control siRNA (NC‐siRNA) to transfect MDA‐MB‐231 cells. The transfection efficiency was verified by qRT‐PCR (Figure 4B). Western blot analysis results showed that compared with transfection with NC‐siRNA, EZH2 protein expression was significantly down‐regulated after transfection with EZH2 siRNA, whereas DLC1 expression was up‐regulated (Figure 4C). To further verify that EZH2 can inhibit the expression of DLC1 by mediating H3K27me3, we used EZH2 siRNA1 to transfect 231 cells and found that EZH2 knockdown can inhibit the expression of H3K27me3 protein (Figure 4D). ChIP assays showed that H3K27me3 was enriched in the DLC1 promoter region of MDA‐MB‐231 cells, while the knockdown of EZH2 reduced the enrichment of H3K27me3 in the promoter region of DLC1 (Figure 4E). These results suggest that knockdown of EZH2 can restore the expression of DLC1 by reducing the enrichment of H3K27me3 in the DLC1 promoter.

### 3.5 Both EZH2 and DLC1 are related to genes involved in cell proliferation, apoptosis and the cell cycle in BC

The correlation analysis based on TCGA demonstrated that the expression of EZH2 was positively correlated with the expression of MKI67 (*R* = 0.64, *P* = 0, Figure 4F) and CDK1 (*R* = 0.51, *P* = 0, Figure 4G) and negatively correlated with the expression of CASP9 (*R* = −0.15, *P* = 1.5 × 10^−6^, Figure 4H) in BC. However, the expression of DLC1 was negatively correlated with the expression of MKI67 (*R* = −0.099, *P* = 0.001, Figure 4I) and CDK1 (*R* = −0.14, *P* = 5.9 × 10^−6^, Figure 4J) and positively correlated with the expression of CASP9 (*R* = 0.19, *P* = 2.6 × 10^−10^, Figure 4K) in BC.

### 3.6 EZH2 inhibitor and knockdown of EZH2 can inhibit the migration, invasion and proliferation of TNBC cells, promote apoptosis and block the cell cycle

**FIGURE 5:**
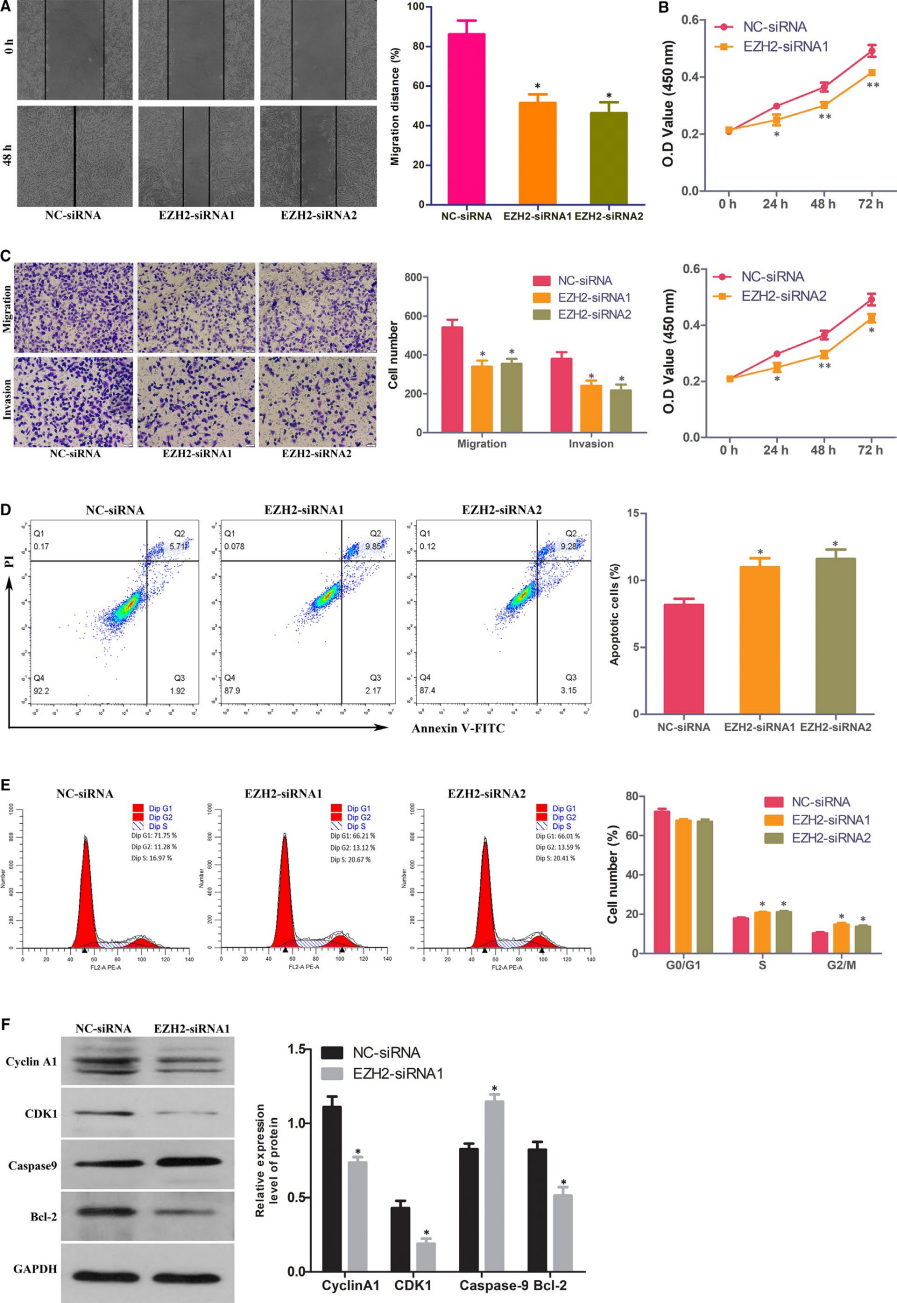
Knocking down EZH2 inhibits MDA‐MB‐231 cell proliferation, migration and invasion, promotes cell apoptosis and blocks the cell cycle. A, Wound healing assays showed that knockdown of EZH2 inhibits MDA‐MB‐231 cell migration. B, Proliferation of MDA‐MB‐231 cells transfected with EZH2 siRNA (EZH2‐siRNA) or negative control siRNA (NC‐siRNA) was detected by CCK‐8 assay. C, Transwell assays showed that knockdown of EZH2 decreased MDA‐MB‐231 cell migration and invasion. D, Knockdown of EZH2 increased the apoptosis of MDA‐MB‐231 cells. E, The cell cycle was analysed in MDA‐MB‐231 cells transfected with EZH2‐siRNA or NC‐siRNA. F, Expression of cyclin A1, CDK1, caspase‐9 and Bcl‐2 protein in MDA‐MB‐231 cells transfected with EZH2‐siRNA or NC‐siRNA. *P < 0.05; **P < 0.01

To further determine the effects of EZH2 on TNBC cells, cell proliferation, apoptosis, cell cycle, migration and invasion assays were performed. Transwell and wound healing assays showed that knockdown of EZH2 by transfection with EZH2 siRNA inhibited the invasion and migration of MDA‐MB‐231 cells (Figure 5A,C) and MDA‐MB‐468 cells (Figure S1). Moreover, the results of the CCK‐8 assay demonstrated that knockdown of EZH2 inhibited the proliferation of MDA‐MB‐231 cells (Figure 5B) and MDA‐MB‐468 cells (Figure S1). To explore how EZH2 regulated cell proliferation, we detected the apoptosis and cycle of MDA‐MB‐231 cells and MDA‐MB‐468 cells by flow cytometry. The results showed that knocking down EZH2 by EZH2 siRNA increased the apoptosis of MDA‐MB‐231 cells (Figure 5D) and MDA‐MB‐468 cells (Figure S1) and blocked MDA‐MB‐231 cells (Figure 5E) and MDA‐MB‐468 cells (Figure S1) in G2 phase compared with NC‐siRNA. To confirm this result, apoptosis‐ and cell cycle‐related proteins were examined. Western blot analysis (Figure 5F) demonstrated that EZH2 siRNA decreased cyclin A1 CDK1 and Bcl‐2 protein levels and increased caspase‐9 protein levels in MDA‐MB‐231 cells. In addition, we used EZH2 inhibitors to treat MDA‐MB‐231 cells and MDA‐MB‐468 cells and found that the EZH2 inhibitor can inhibit the migration, invasion and proliferation of MDA‐MB‐231 cells and MDA‐MB‐468 cells, promote apoptosis and block the cell cycle (Figure S2).

### 3.7 Curcumin inhibits the migration, invasion and proliferation of TNBC cells, promotes apoptosis and blocks the cell cycle

**FIGURE 6:**
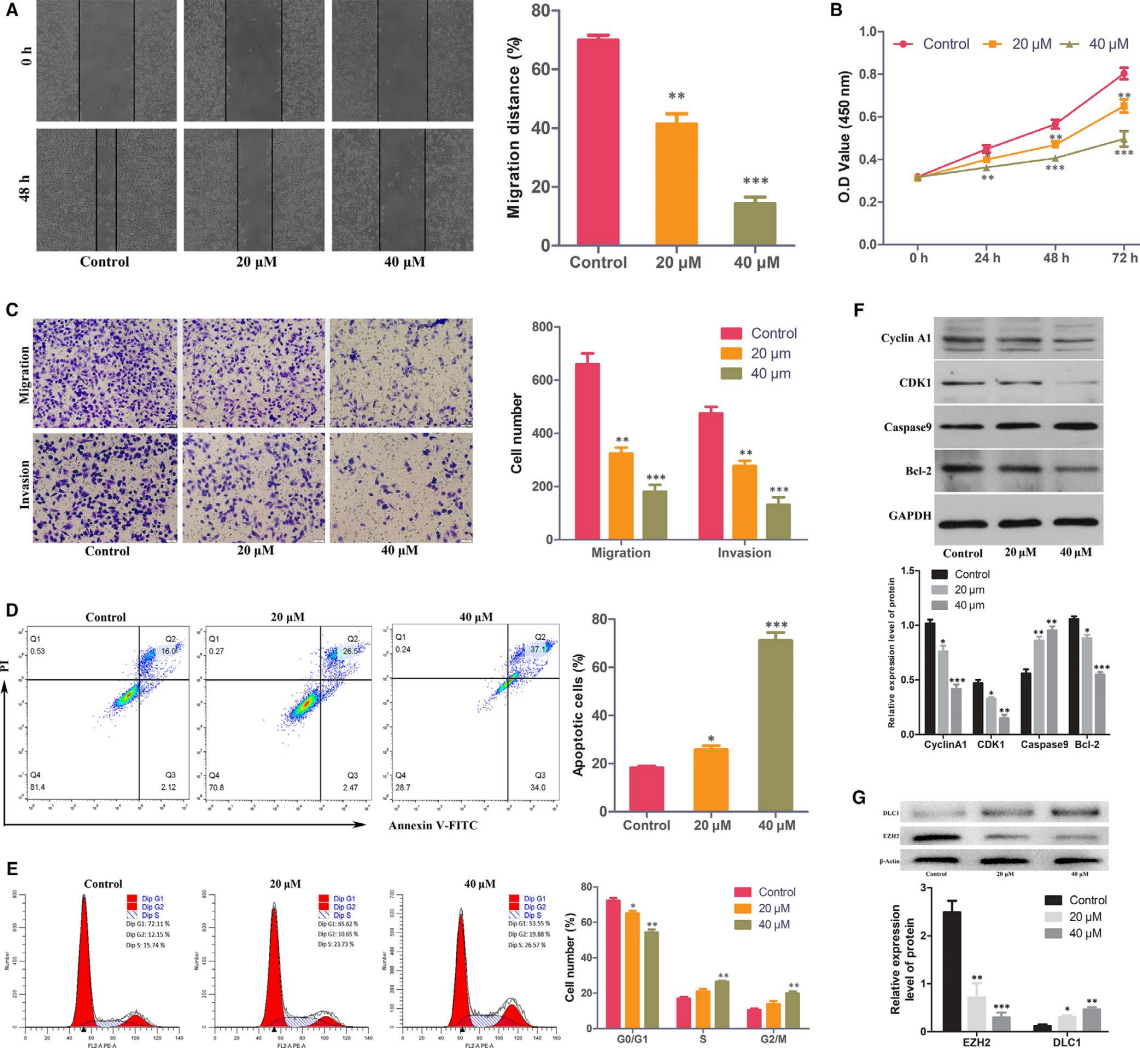
Curcumin inhibits MDA‐MB‐231 cell proliferation, migration and invasion, promotes cell apoptosis and blocks the cell cycle. A, Wound healing assays showed that curcumin inhibited MDA‐MB‐231 cell migration. B, Curcumin inhibited the proliferation of MDA‐MB‐231 cells. C, Transwell assays showed that curcumin decreased MDA‐MB‐231 cell migration and invasion. D, Curcumin increased the apoptosis of MDA‐MB‐231 cells. E, Curcumin blocked the MDA‐MB‐231 cell cycle. F, Expression of cyclin A1, CDK1, caspase‐9 and Bcl‐2 protein in MDA‐MB‐231 cells treated with 20, 40 μmol/L curcumin or control. G, Expression of EZH2 and DLC1 protein in MDA‐MB‐231 cells treated with curcumin or control. *P < 0.05; **P < 0.01; ***P < 0.001

To confirm the effect of curcumin on TNBC cells, we also performed migration cell apoptosis, cell cycle, proliferation, invasion and migration assays. Transwell and wound healing assays showed that curcumin could inhibit the invasion and migration of MDA‐MB‐231 cells (Figure 6A,C) and MDA‐MB‐468 cells (Figure S3). Moreover, the results of the CCK‐8 assay demonstrated that the proliferation of MDA‐MB‐231 cells (Figure 6B) and MDA‐MB‐468 cells (Figure S3) was inhibited by curcumin. To explore how curcumin regulated cell proliferation, we detected the apoptosis and cell cycle distribution of MDA‐MB‐231 cells and MDA‐MB‐468 cells using flow cytometry. The results demonstrated that compared to the control, curcumin increased the apoptosis of MDA‐MB‐231 cells (Figure 6D) and MDA‐MB‐468 cells (Figure S3) and blocked MDA‐MB‐231 cells (Figure 6E) and MDA‐MB‐468 cells (Figure S3) in G2 phase. To confirm this result, apoptosis‐ and cell cycle‐related proteins were examined. WB analysis (Figure 6F) demonstrated that curcumin decreased cyclin A1, CDK1 and Bcl‐2 protein levels and increased caspase‐9 protein levels in MDA‐MB‐231 cells.

### 3.8 Curcumin restores the expression of DLC1 by inhibiting EZH2 in MDA‐MB‐231 cells

To demonstrate the effect of curcumin on EZH2 and DLC1, WB analysis was carried out. The results showed that the expression of EZH2 in MDA‐MB‐231 cells was significantly down‐regulated after curcumin treatment, while the expression of DLC1 was up‐regulated (Figure 6G).

### 3.9 Curcumin inhibits the growth of TNBC and restores the expression of DLC1 by inhibiting EZH2 in vivo

**FIGURE 7:**
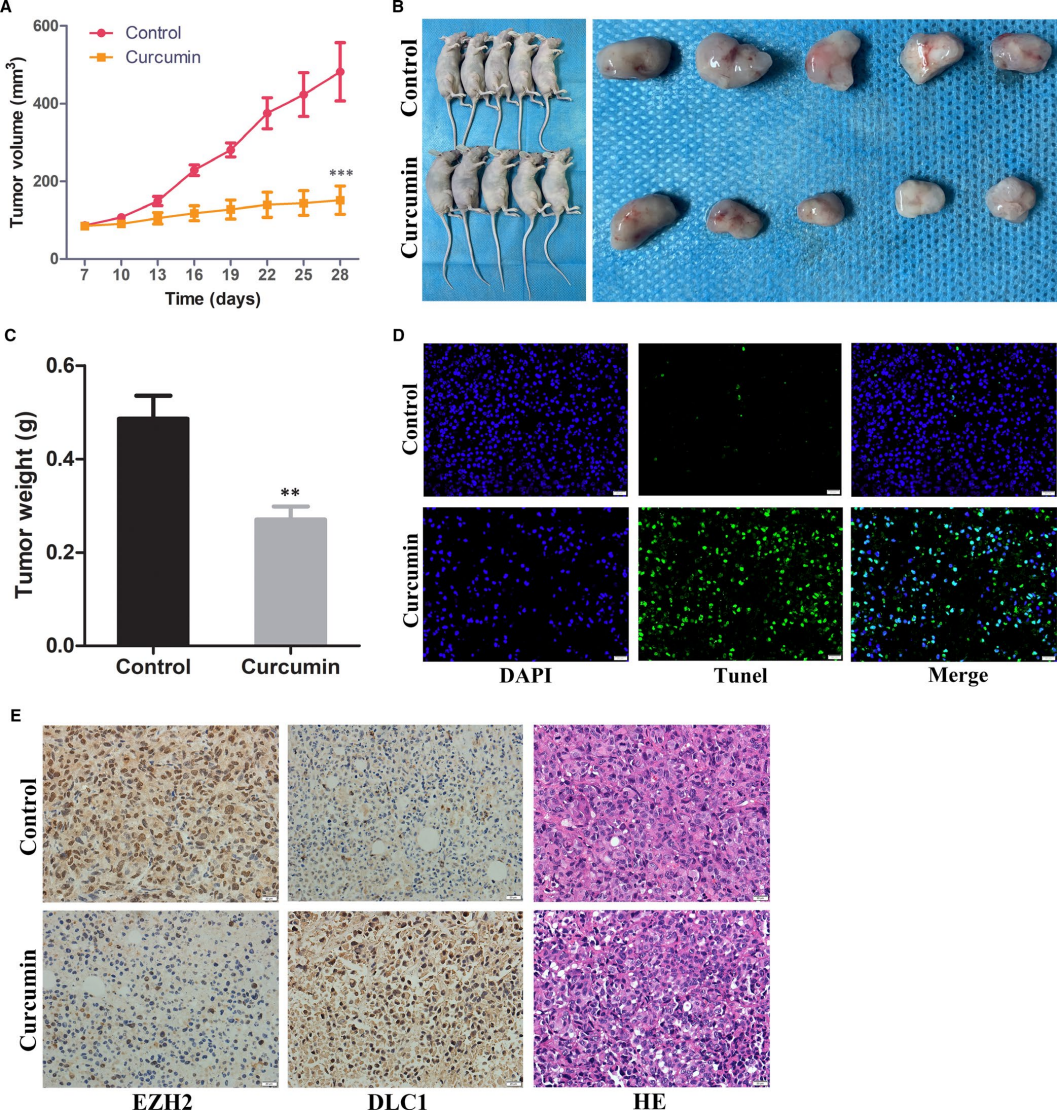
Curcumin inhibits the growth of triple‐negative breast cancer cells by inhibiting EZH2 expression and reversing DLC1 expression in vivo. A, The changes in the average volume of tumours were measured (n = 10). B, The xenograft tumours were removed after 4 wk of cell injection. C, Tumour weight in different groups after 28 d. D, Detection of apoptosis in xenograft tumour tissues by TUNEL assay (×400, bar = 20 μm). E, Representative pictures of EZH2 and DLC1 immunohistochemical (IHC) staining in xenograft tumour tissues. (×400, bar = 20 μm, n = 10). **P < 0.01; ***P < 0.001

Finally, we assessed the effect of curcumin on the growth of TNBC cell lines by a xenograft model. The model was established by injecting MDA‐MB‐231 cells into nude mice and then treating them with curcumin for 21 days. The results showed that curcumin inhibited the growth of tumours (Figure 7B). Moreover, both volume and weight measurements demonstrated that curcumin significantly inhibited the growth of tumours (Figure 7A,C). In addition, TUNEL results demonstrated that the apoptotic rate of xenograft tumour tissue increased after curcumin treatment (Figure 7D). IHC showed that the expression of EZH2 was down‐regulated and the expression of DLC1 was up‐regulated in TNBC xenograft tumour tissues treated with curcumin (Figure 7E).

## 4 DISCUSSION

As a common malignant tumour in women, the occurrence and development of BC are a complex process of genetic and epigenetic changes. Epigenetics is a hereditary gene expression regulation mode that does not involve DNA sequence changes, including histone modification, gene imprinting and DNA methylation.^22^

EZH2 is a core member of the PRC2 gene silencing complex that has methyltransferase activity and catalyses histone H3K27 trimethylation and heterochromatin formation, and thereby mediates tumour suppressor gene expression silencing.^23^ The overexpression of EZH2 can occur in many cancers, including endometrial cancer, prostate cancer, melanoma, bladder cancer and BC.^24^, ^25^ Moreover, some studies^10^, ^26^, ^27^, ^28^ have shown that overexpression of EZH2 is correlated with poor prognosis in many cancers. Similarly, our study also found that EZH2 is overexpressed in BC and is correlated with the size of tumours, lymph node metastasis and poor prognosis of TNBC patients. In this study, we identified that EZH2 and DLC1 were negatively correlated in BC based on TCGA database analysis (*R* = −0.13, *P* = 2.7 × 10^−5^) and further confirmed that EZH2 and DLC1 were negatively correlated in BC patients by collecting clinical samples (*R* = −0.248, *P* = 0.013). We also found that EZH2 and DLC1 were correlated with genes involved in cell apoptosis, cell cycle progression and proliferation in BC. Functional assays further proved that knocking down EZH2 inhibited the invasion, migration and proliferation of MDA‐MB‐231 cells, promoted cell apoptosis and blocked the cell cycle. Moreover, some studies^29^, ^30^, ^31^ have also demonstrated that EZH2 can promote the invasion and proliferation of tumour cells.

As a candidate antioncogene, DLC1 is easily inactivated or lost in HCC.^13^ It encodes three main functional domains: RhoGAP, C‐terminal START and N‐terminal SAM. Rho proteins participate in the regulation of normal physiological processes, such as actin cytoskeleton composition, cell movement, proliferation and transcription. However, abnormal activation of Rho protein can lead to tumorigenesis and metastasis.^32^ RhoGAP can increase the activity of endogenous GTP of Rho family proteins and hydrolyse GTP to GDP, thus changing Rho family proteins from the active form to the inactive form.^33^ In many cancer cells, the inactivation of RhoGAP caused by DLC1 gene silencing can activate RhoGAP protein, which can continuously transmit growth signals to cells, which may be one of the main mechanisms of tumorigenesis.^34^ However, inactivation of the DLC1 gene occurs not only in liver cancer^35^ and lung cancer,^36^ but its low expression or loss of expression in gastric cancer, BC and prostate cancer results in its malignant phenotype.^37^ Some studies have confirmed that DLC1 can inhibit the migration and proliferation of HCC cells^38^ and BC cells.^39^, ^40^ Our study found that knocking down EZH2 up‐regulated the expression of DLC1, which may be another reason why knocking down EZH2 promotes the apoptosis of MDA‐MB‐231 cells and inhibits their migration and proliferation.

Curcumin, as a promising anticancer drug, has antitumour properties in many tumours, including BC.^41^, ^42^, ^43^ Wenfeng Hua et al^44^ found that curcumin inhibited the proliferation of MDA‐MB‐435 BC cells by down‐regulating the expression of EZH2. To further demonstrate the role of curcumin in TNBC, we treated MDA‐MB‐231 cells with curcumin. The results show that curcumin inhibited cell migration, invasion and proliferation, promoted cell apoptosis and blocked the cell cycle. Moreover, we found that the expression of EZH2 was down‐regulated and DLC1 was up‐regulated after treatment of MDA‐MB‐231 cells with curcumin. This indicates that curcumin may restore the expression of DLC1 by down‐regulating the expression of EZH2, thereby promoting apoptosis and inhibiting the proliferation and metastasis of MDA‐MB‐231 cells.

This study explored the expression of EZH2 and DLC1 in BC and assessed their regulatory relationship in TNBC. In addition, we explored the role of curcumin in TNBC and its possible mechanism of action. In summary, we demonstrated that the expression of EZH2 is up‐regulated in BC, the expression of DLC1 is down‐regulated in BC, and both up‐regulation of EZH2 and down‐regulation of DLC1 are correlated with poor prognosis in TNBC patients. Both of them may be prognostic biomarkers and potential therapeutic targets for TNBC. Moreover, we also found that curcumin can repress the growth of MDA‐MB‐231 cells, and this process may be achieved by restoring the expression of DLC1 and inhibiting the expression of EZH2. Finally, we further validated the effect of curcumin in vivo and obtained the same results. This also provides a reference for the treatment of BC with curcumin.

## CONFLICT OF INTEREST

All authors confirm that there are no conflicts of interest.

## AUTHOR CONTRIBUTION

**Xueliang Zhou:** Conceptualization (lead); Data curation (lead); Methodology (lead); Software (lead); Writing‐original draft (lead). **Dechao Jiao:** Conceptualization (equal); Data curation (equal); Methodology (equal); Resources (equal); Writing‐review & editing (lead). **Mengmeng Dou:** Data curation (equal); Methodology (equal); Writing‐review & editing (equal). **Weijie Zhang:** Data curation (equal); Resources (equal). **Liying Lv:** Data curation (equal). **Jianjian Chen:** Software (equal). **Lifeng Li:** Methodology (equal). **Liuxing Wang:** Resources (equal). **Xinwei Han:** Conceptualization (equal).

## Supporting information

Fig S1

Fig S2

Fig S3

## Data Availability

All data from this study are available and have been shown in the article.
