# Supplementary figures and images for: Curcumin inhibits the growth of triple‐negative breast cancer cells by silencing EZH2 and restoring DLC1 expression
Source: J Cell Mol Med. 2020 Jul 28;24(18):10648–62. doi: 10.1111/jcmm.15683 (PMC7521266; doi:10.1111/jcmm.15683)

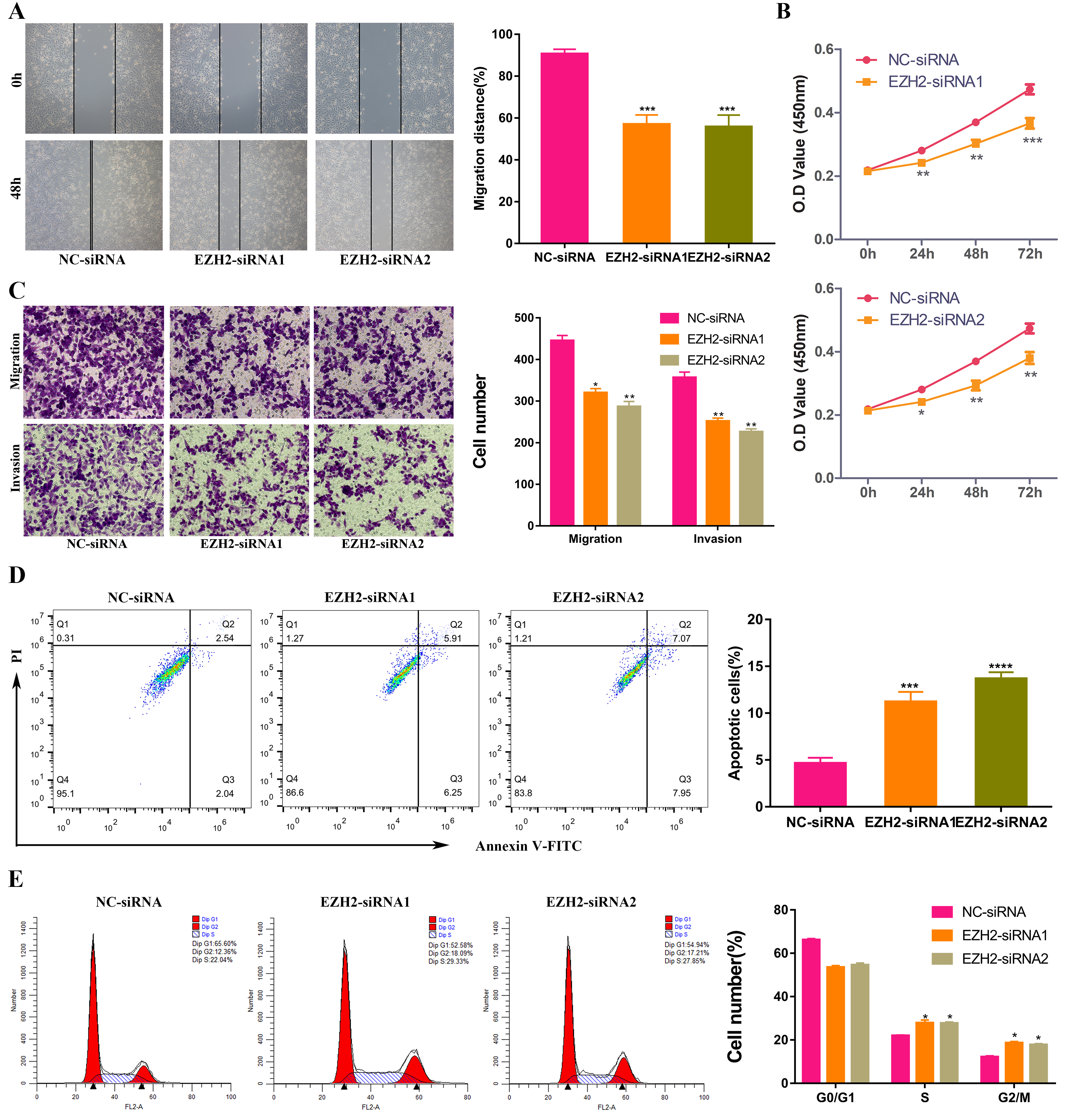

Supplement: Supplementary file 1 — Fig S1 [file JCMM-24-10648-s001.tif]

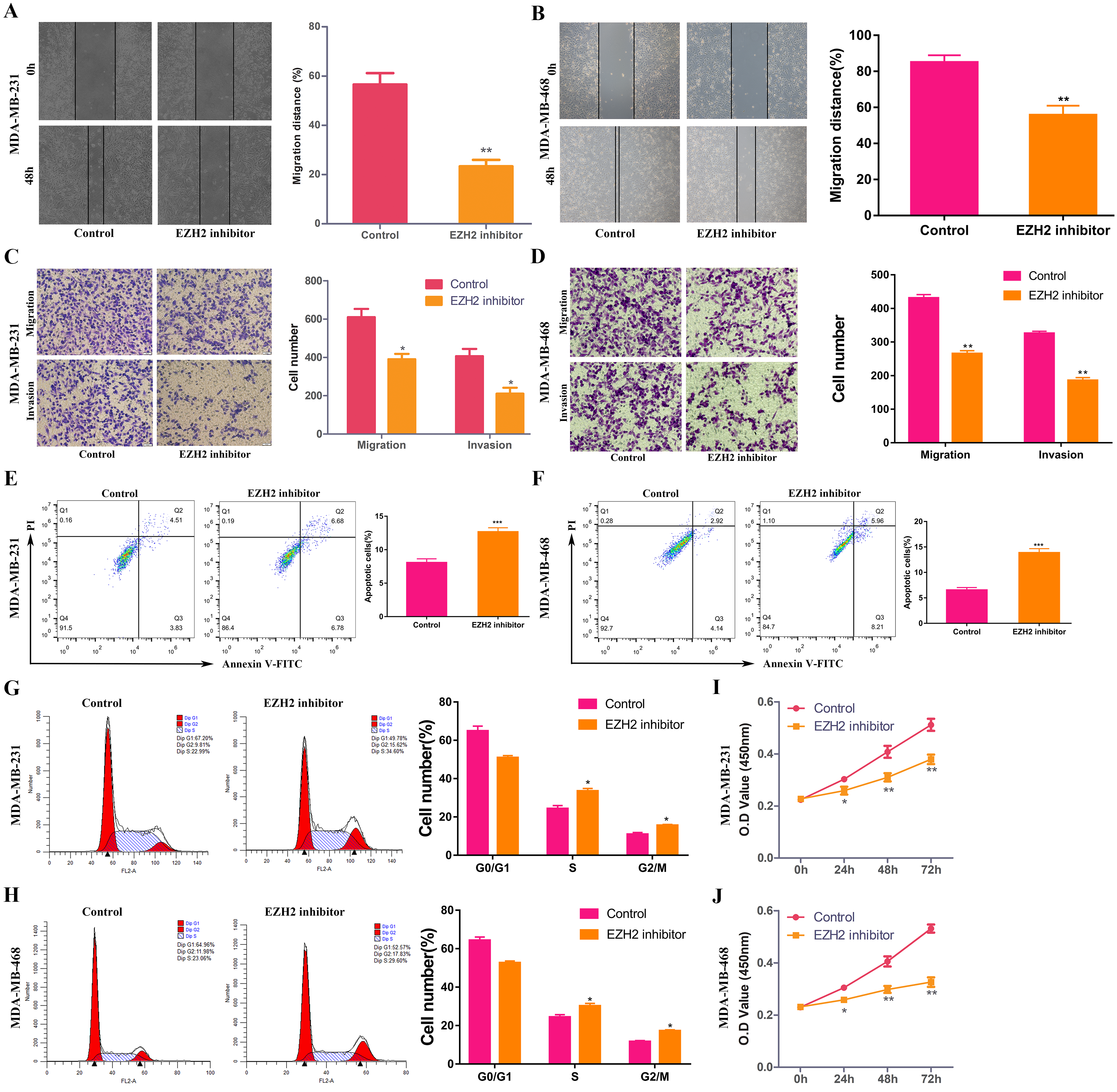

Supplement: Supplementary file 2 — Fig S2 [file JCMM-24-10648-s002.tif]

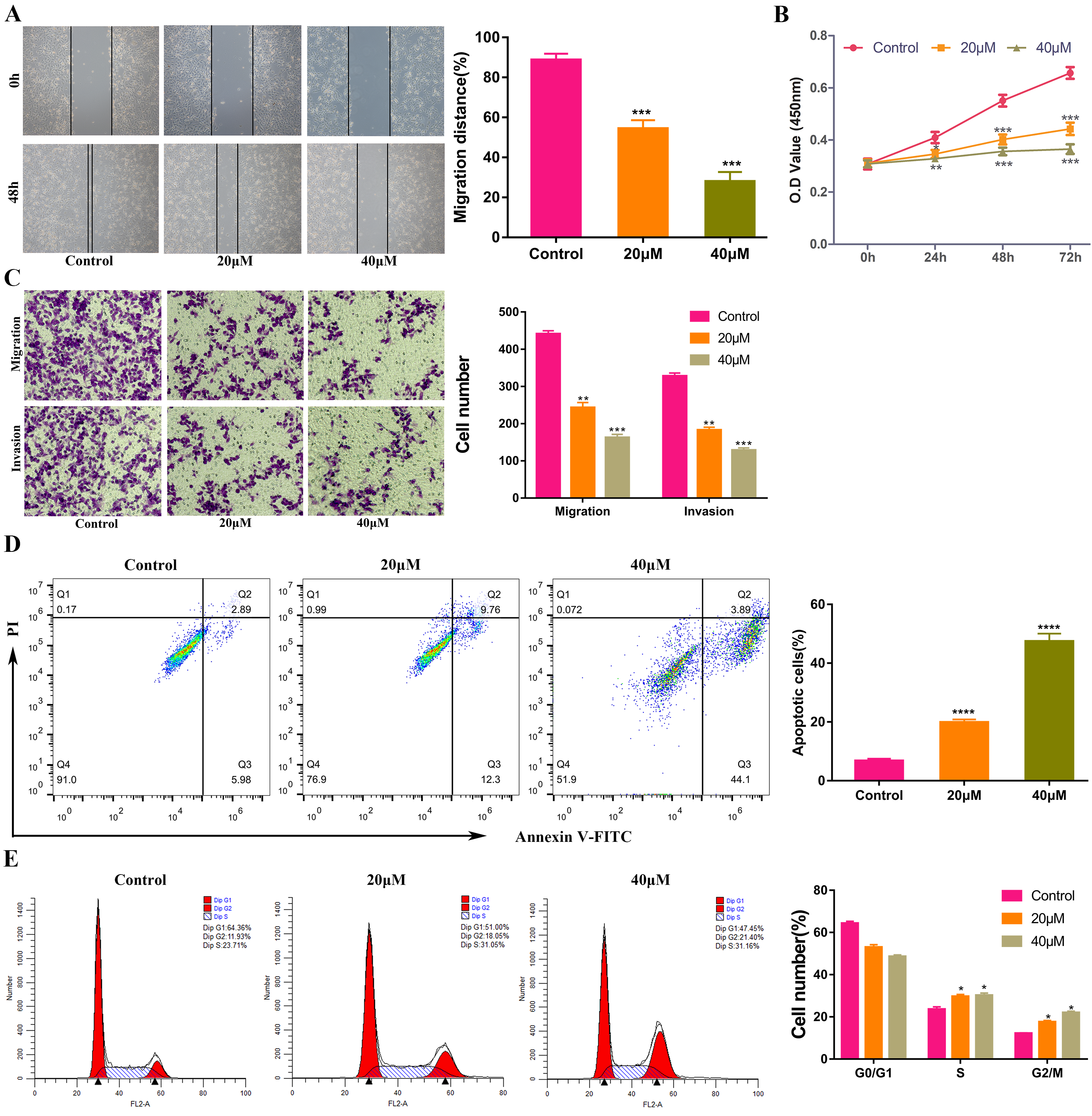

Supplement: Supplementary file 3 — Fig S3 [file JCMM-24-10648-s003.tif]
